# The downregulation of tight junction proteins and pIgR in the colonic epithelium causes the susceptibility of EpCAM^+/−^ mice to colitis and gut microbiota dysbiosis

**DOI:** 10.3389/fmolb.2024.1442611

**Published:** 2024-08-12

**Authors:** Ya Nie, Ting Lin, Yanhong Yang, Wanwan Liu, Qing Hu, Guibin Chen, Li Huang, Huijuan Wu, Cunjie Kong, Zili Lei, Jiao Guo

**Affiliations:** ^1^ Guangdong Metabolic Diseases Research Center of Integrated Chinese and Western Medicine, Key Laboratory of Glucolipid Metabolic Disorder, Ministry of Education of China, Guangdong Pharmaceutical University, Guangzhou, China; ^2^ School of Traditional Chinese Medicine, Guangdong Pharmaceutical University, Guangzhou Higher Education Mega Center, Guangzhou, China; ^3^ The First Affiliated Hospital, School of Clinical Medicine, Guangdong Pharmaceutical University, Guangzhou, China

**Keywords:** EpCAM, inflammatory bowel disease, tight junction, pIgR, gut microbiota

## Abstract

**Background:**

The genetic factors play important roles on the pathogenesis of inflammatory bowel disease (IBD). EpCAM is highly expressed in the intestinal epithelium. It is still unclear if the decrease or somatic mutation of EpCAM could cause IBD.

**Methods:**

The WT and EpCAM^+/−^ mice were administrated with DSS intermittently for nearly 8 weeks. The colon, liver and feces were harvested to check the morphological and histological changes, the expression of inflammatory genes and the gut microbiota via H&E staining, immunofluorescence, qPCR, western blot and 16S rDNA sequence assays.

**Results:**

The DSS administration induced more serious inflammation in the colon of EpCAM^+/−^ mice than WT mice. Compared to DSS-induced WT mice, the transcriptional levels of IL-6, F4/80, Ly6g, Ly6d and Igha were significantly higher in the colon of DSS-induced EpCAM^+/−^ mice. The protein levels of MMP7 and MMP8 and the activation of JNK, ERK1/2 and p38 were significantly increased in the colon of DSS-induced EpCAM^+/−^ mice. The protein levels of CLDN1, CLDN2, CLDN3, CLDN7, OCLD, ZO-1 and pIgR were significantly decreased in the colon of DSS-induced EpCAM^+/−^ mice. The serum concentration of LPS was significantly higher in the DSS-induced EpCAM^+/−^ mice which caused the acute inflammation in the liver of them. The expression of Pigr was significantly reduced in the liver of DSS-induced EpCAM^+/−^ mice. The ratio of *Firmicutes*/*Bacteroidetes* at the phylum level was higher in the gut microbiota of EpCAM^+/−^ mice than WT mice.

**Conclusion:**

In conclusion, the heterozygous mutation of EpCAM increased the susceptibility to colitis, gut microbiota dysbiosis and liver injury.

## 1 Introduction

EpCAM is highly localized in the basolateral membrane of intestinal epithelial cells (IECs) (Huang et al., 2018). The congenital tufting enteropathy (CTE) which is a rare disease of human is caused by the homozygous mutation of EpCAM (Sivagnanam et al., 2008). The inflammation has been detected in the intestines of CTE patients and EpCAM^−/−^ mice (Gerada et al., 2013; Lei et al., 2022), indicating the deficiency of EpCAM leads to the breakdown of the intestinal immune homeostasis. CTE has been considered as one kinds of very early onset IBD (Kammermeier et al., 2014). The knockdown of EpCAM in the colon increases the severity of DSS-induced IBD of mice (Jiang et al., 2016). The parents of CTE patients should be the heterozygotes of EpCAM mutation. It is still unclear if the intestinal immune homeostasis of the heterozygotes of EpCAM mutation would be affected.

The apical and the basolateral plasma membrane domains of IECs are separated by the network of tight junctions which seal the adjacent epithelial cells (Tsukita et al., 2001). The knockout of JAM-A, one of the key structural components of tight junctions, causes mild colitis in mice, and these mice show the increased susceptibility to DSS induced IBD (Laukoetter et al., 2007, Serek and Oleksy-Wawrzyniak, 2021). The deficiency of tight junction protein Claudin 7 also causes serious inflammation in the intestines (Ding et al., 2012). Therefore, the tight junctions in the intestinal epithelium are essential to maintain the immune homeostasis of intestines. EpCAM has important roles on maintaining the normal physiological function of tight junctions in the intestinal epithelium (Lei et al., 2012). Hence, the tight junctions in the intestinal epithelium of the heterozygotes of EpCAM mutation still need to be explored.

The immunoglobulin A (IgA) and IgM have important function on the intestinal immune protection (Chen et al., 2020; Casali et al., 2021). They are generated by plasma cells in the lamina propria and then are translocated across intestinal epithelium by pIgR (polymeric immunoglobulin receptor) (Chen et al., 2020). Part of IgA antibodies are translocated across hepatocytes from blood into the bile through pIgR and then are transported into the intestinal lumen (Shimada et al., 1999). Somatic mutation of PIGR has been detected in inflamed intestinal epithelium of patients with ulcerative colitis (UC) (Nanki et al., 2020). It has also been reported that knockout of pIgR exacerbates DSS-induced colitis (Murthy et al., 2006). pIgR is significantly downregulated in the intestinal epithelium of EpCAM^−/−^ mice (Lei et al., 2022). However, it is still unclear if the expression of pIgR would be affected in the intestines of heterozygotes of EpCAM mutations.

The host-gut microbiota crosstalk has been reported to have important functions in the physiology and disease (Marchix et al., 2018; Ma et al., 2022). The miRNAs from the host with IBD has been demonstrated to shape the gut microbiota (Casado-Bedmar et al., 2022). The gut microbiota derived lipopolysaccharide (LPS) has been found to exacerbate the progression of acute myeloid leukaemia because of the impaired tight junctions in the intestinal epithelium (Wang R. et al., 2022). Many studies have demonstrated that LPS can induce the liver inflammation or even liver injury (Wang L. et al., 2022; Zhang et al., 2022). However, the crosstalk between the heterozygotes of EpCAM mutations and gut microbiota of them is still unclear.

In the present study, EpCAM^+/−^ mice were administrated with DSS to explore the susceptibility and related mechanisms of the heterozygotes of EpCAM mutations to IBD. Our work demonstrated that the heterozygous mutation of EpCAM might be one of the genetic factors leading to IBD and the gut microbiota might also play important roles in the occurrence of IBD in the heterozygotes of EpCAM mutations.

## 2 Materials and methods

### 2.1 Mice

**FIGURE 1:**
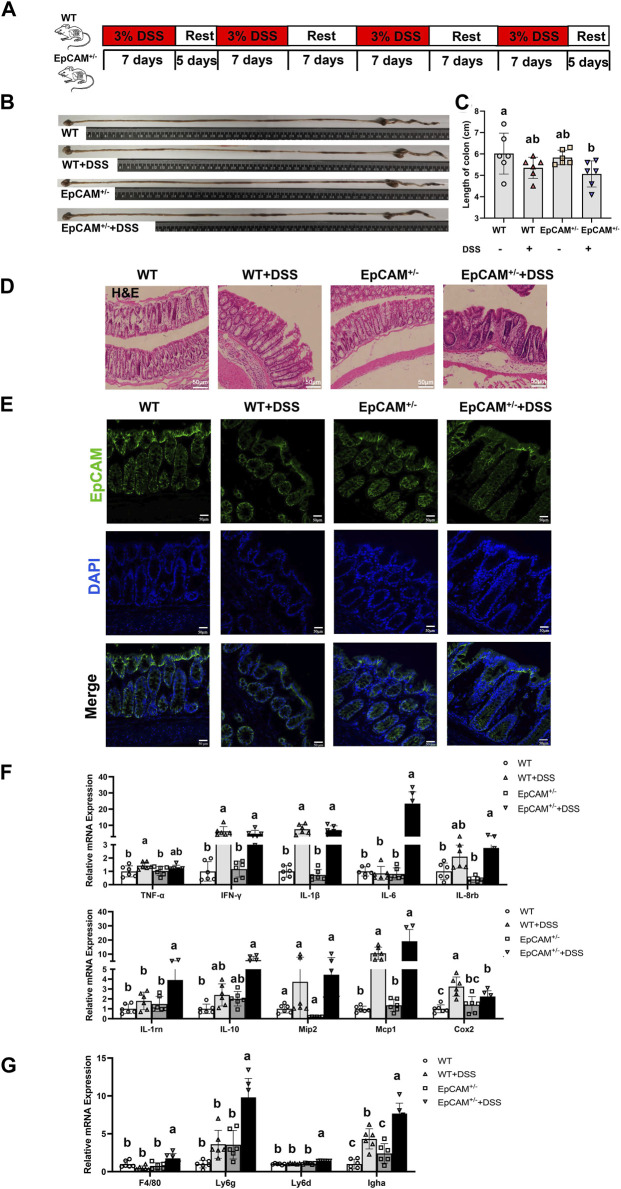
DSS induced more serious inflammation in the colon of EpCAM^+/−^ mice **(A)**. The scheme of the experimental design **(B)**. Representative images of intestines from WT, WT + DSS, EpCAM^+/−^ and EpCAM^+/−^ + DSS groups **(C)**. Graph showed the length of large intestines from WT, WT + DSS, EpCAM^+/−^ and EpCAM^+/−^ + DSS groups **(D)**. Representative images of H&E staining of paraffin sections of colons from mice of WT, WT + DSS, EpCAM^+/−^ and EpCAM^+/−^ + DSS groups. Scale bar, 50 μm **(E)**. Representative images of immunofluorescence staining with antibodies to EpCAM of frozen sections of colon from mice of WT, WT + DSS, EpCAM^+/−^ and EpCAM^+/−^ + DSS groups. DAPI was used for counter staining. Scale bar, 50 μm **(F)**. Graphs showed relative mRNA expression levels of TNFα, IFN-γ, IL-1β, IL-6, IL-8rb, IL-1rn, IL-10, Mip2, Mcp1 and Cox2 in the colon of mice from WT, WT + DSS, EpCAM^+/−^ and EpCAM^+/−^ + DSS groups **(G)**. Graphs showed the qPCR results of F4/80, Ly6g, Ly6d and Igha in the colon of mice from WT, WT + DSS, EpCAM^+/−^ and EpCAM^+/−^ + DSS groups. a, b, c, meaning significant difference among groups. WT, wild type (EpCAM^+/+^); DSS, dextran sulfate sodium salt; TNFα, tumor necrosis factor alpha; IFN-γ, interferon gamma; IL-1β, interleukin 1 beta; IL-6, interleukin 6; IL-8rb, interleukin-8 receptor B; IL-1rn, interleukin 1 receptor antagonist; IL-10, interleukin 10; Mip2, macrophage inflammatory protein-2; Mcp1, monocyte chemoattractant protein-1; Cox2, cyclooxygenase 2; F4/80, cell surface glycoprotein F4/80; Ly6g, lymphocyte antigen 6 complex, locus G; Ly6d, lymphocyte antigen 6 complex, locus D; Igha, immunoglobulin heavy constant alpha.

All the animal experiments were approved by the Experimental Animal Ethics Committee of Guangdong Pharmaceutical University and adhered to the ARRIVE guidelines. The 15–17 weeks old female WT and EpCAM^+/−^ mice were used, and they were got from mating the C57BL/6 WT females with the EpCAM^+/−^ males which were previously generated (Yang et al., 2019). Mice were housed in the SPF mouse facility, at 25°C, 12 h light-dark cycle, 60%–65% humidity, with free access to water and food. The mice were divided into WT, WT + DSS, EpCAM^+/−^ and EpCAM^+/−^ + DSS groups, 6 mice in each group. For the WT + DSS and EpCAM^+/−^ + DSS groups, mice drank waters containing 3% DSS (CD4421-100 g, Coolaber) *ad libitum* according to the procedures shown in Figure 1A.

### 2.2 Hematoxylin and eosin (H&E) staining

The colonic tissues were washed quickly with cold PBS, and then were transferred into the fixative of 4% paraformaldehyde in PBS. After the overnight fixation at 4°C, the tissues were dehydrated with ethanol series, then were embedded in paraffin and sectioned to 4-µm-thick sections. After dewaxing, the sections were stained with hematoxylin (H9627, Sigma-Aldrich) for 3 min at room temperature, and then were treated with eosin (E4009, Sigma-Aldrich) for 20 s. The images of H&E staining were taken with the Olympus DP74 microscope.

### 2.3 Immunofluorescence (IF) staining

The IF staining was conducted as previously described (Lei et al., 2021b). The primary antibodies included rabbit anti-EpCAM (1:100; Cat. No. ab71916) and rat anti-pIgR (1:400; Cat. No. ab170321; Abcam). The Alex Fluor 488-labeled secondary antibodies (Invitrogen) were used for the analysis. Images were got through the Olympus confocal microscope.

### 2.4 qRT-PCR

To extract total RNA, the dissected colonic tissues were washed in cold PBS quickly, and then were treated using Trizol reagent (Invitrogen; Thermo Fisher Scientific, Inc.). The RNA quality and concentration were checked through Nano Drop. Then, the cDNA was synthesized using the PrimeScript™ RT Reagent kit (Takara Bio, Inc.) according to the manufacturer’s protocol. The qPCR was carried out using the SYBR Premix Ex Taq kit (Takara Bio, Inc.) and the LightCycler 480II System (Roche, Inc.). The mouse GAPDH was used as the reference for the calculation of the mRNA relative expression. All primers used for qPCR were listed in Supplementary Table S1.

### 2.5 Western blot

The colonic tissues were washed quickly in cold PBS, and then lysed using the Radio-Immunoprecipitation Assay lysis buffer (Dalian Meilun Biotechnology co., Ltd.). Lysates were cleared through centrifugation at 13,680 × g, at 4°C, for 30 min. The protein concentration was determined using BCA kit (P0011, Beyotime). Then, 30 μg protein was separated through the sodium dodecyl sulfate polyacrylamide gel electrophoresis, and then the separated proteins were transferred electrophoretically to the PVDF membrane. The PVDF membrane was exposed in the TBST buffer containing 10% skimmed milk at room temperature for 1 h, then was probed with primary antibodies at 4°C for overnight. After washing and incubation with HRP (horseradish peroxidase)-labeled secondary antibodies, the blots were detected using enhanced chemiluminescence reagent (Bio-Rad 170–5060). The densitometry was analyzed relative to the GAPDH loading control using the ImageJ software (version 1.53a). The primary and secondary antibodies used for WB were listed in Supplementary Table S2.

### 2.6 Determination of inflammatory factors in serum

The concentrations of LPS, TNF-α and IL-1β in the serum were measured according to the manufacturer’s protocol for each kit. The determination kits for LPS (CSB-E13066 m), TNF-α (CSB-E04741 m) and IL-1β (CSB-E08054 m) were purchased from Nanjing Jiancheng Bioengineering Institute (Nanjing, China).

### 2.7 16S rDNA gene analysis

Fecal samples of mice were quickly frozen in liquid nitrogen and then stored at −80°C. Fecal bacterial DNA extraction, 16S rDNA gene PCR amplification, sequencing, and analysis were performed by Gene *Denovo* Biotechnology Company (Guangzhou, China). The procedures were described previously (Lei et al., 2021a).

### 2.8 Statistical analysis

Statistical analysis was determined via the SPSS software (version 23.0; IBM Corp.). Mean ± SEM was used to express data. One-way ANOVA followed by Tukey’s *post hoc* test was conducted to analyze differences among the groups, and the *p*-value <0.05 was considered to be significant.

## 3 Results

### 3.1 The heterozygous mutation of EpCAM increased the sensitivity to the DSS-induced colitis

To explore the impact of the heterozygous mutation of EpCAM on the intestinal immune homeostasis, the WT and EpCAM^+/−^ mice were administrated with 3% DSS to induce the colitis as shown in Figure 1A. After administration, the average length of the colon in the EpCAM^+/−^ + DSS mice was the shortest among 4 groups, and importantly, it was significantly shorter than the WT group (*p < 0.05*) (Figures 1B,C). The DSS administration caused the infiltration of inflammatory cells in the colon of mice, especially in the EpCAM^+/−^ mice (Figure 1D). The protein level of EpCAM was lower in colons of heterozygotes than the WT mice, and the DSS administration also caused the reduction of EpCAM in the colon especially of WT mice (Figure 1E). These results were consistent with our previous report that the short-term DSS treatment caused the decrease of EpCAM in the colon of mice (Lei et al., 2020). Hence, the downregulation of EpCAM might increase the sensitivity of EpCAM^+/−^ mice to DSS-induced colitis.

To confirm the inflammatory situation in the colon of DSS-induced mice at molecular levels, the transcriptional levels of genes related with inflammatory factors in the colon were subsequently tested via qPCR assay (Figure 1F). The expression of IL-6 was more than 20 folds higher in the colon of EpCAM^+/−^ + DSS group than the WT, WT + DSS and EpCAM^+/−^ groups. The level of TNFα was significantly higher in the WT + DSS group than the WT group (*p < 0.05*), and it was also increased in the EpCAM^+/−^ + DSS group compared to the EpCAM^+/−^ group (*p = 0.275*). The levels of IFN-γ (*p ≤ 0.01*) and IL-1β (*p ≤ 0.01*) were evidently increased in the DSS administrated both WT and EpCAM^+/−^ mice. The levels of IL-8rb and IL-1rn were significantly higher in the EpCAM^+/−^ + DSS group than both WT (*p ≤ 0.01*) and EpCAM^+/−^ (*p < 0.05*) groups, and the mRNA of IL-1rn in EpCAM^+/−^ + DSS group was also noticeably higher than the WT + DSS group (*p ≤ 0.001*). Although the increase of IL-10 in the EpCAM^+/−^ + DSS group was not significant (*p = 0.064*) compared to the EpCAM^+/−^ group, it was significant compared to WT group (*p < 0.05*). The DSS administration also induced the increase of Mip2 in both WT (*p = 0.431*) and EpCAM^+/−^ mice (*p = 0.075*), although not significantly. However, the DSS administration caused more than 10 folds increase of Mcp1 in both WT and EpCAM^+/−^ mice, especially in EpCAM^+/−^ mice. The level of Cox2 was significantly higher in the WT + DSS group than the WT (*p ≤ 0.001*), EpCAM^+/−^ (*p ≤ 0.001*) and EpCAM^+/−^ + DSS (*p < 0.05*) groups, and it was also significantly increased in the EpCAM^+/−^ + DSS group compared to WT mice (*p ≤ 0.01*). The overexpression of the pro-inflammatory cytokine IL-6 in the colon indicated the increased sensitivity of EpCAM^+/−^ mice to DSS-induced colitis compared to WT mice.

Moreover, the mRNA levels of genes related to inflammatory cells in the colon was also checked (Figure 1G). The transcriptional levels of F4/80 (*p < 0.05*), Ly6g (*p ≤ 0.01*), Ly6d (*p ≤ 0.001*) and Igha (*p ≤ 0.001*) were all significantly higher in the EpCAM^+/−^ + DSS group than the WT, WT + DSS and EpCAM^+/−^ groups, and the expressions of Ly6g and Igha increased 5–10 folds in EpCAM^+/−^ + DSS group compared with WT group. The level of Igha was also noticeably increased in WT + DSS group compared to both WT (*p ≤ 0.001*) and EpCAM^+/−^ (*p < 0.05*) groups. These results demonstrated the increase of the infiltrated inflammatory cells and more serious inflammation in the colon of EpCAM^+/−^ + DSS group compared to the WT + DSS mice.

### 3.2 The heterozygous mutation of EpCAM increased the expression of matrix metalloproteinases and the activation of MAPKs in the colon of DSS-induced mice

**FIGURE 2:**
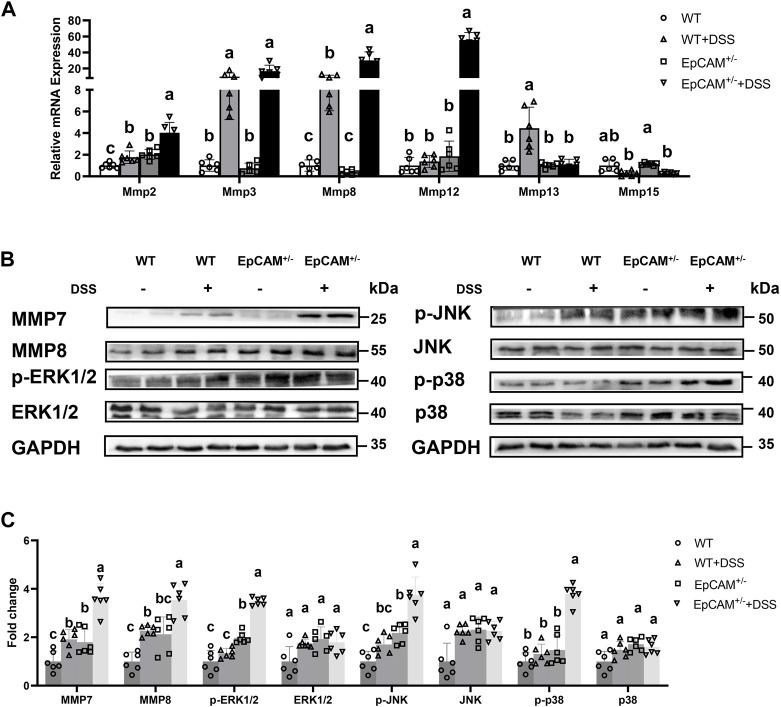
DSS stimulation upregulated the expression of genes for matrix metalloproteinases and hyperactivated MAPK signals in the colon of EpCAM^+/−^ mice **(A)**. The relative mRNA expression levels of Mmp2, Mmp3, Mmp8, Mmp12, Mmp13 and Mmp15 in the colon of mice from WT, WT + DSS, EpCAM^+/−^ and EpCAM^+/−^ + DSS groups **(B)**. Representative images of the western blot results of MMP7, MMP8, p-JNK, JNK, p-ERK1/2, ERK1/2, p-p38 and p38 for the colon of mice from WT, WT + DSS, EpCAM^+/−^ and EpCAM^+/−^ + DSS groups **(C)**. Quantification data of the western blot results of MMP7, MMP8, p-JNK, JNK, p-ERK1/2, ERK1/2, p-p38 and p38 for the colon of mice from WT, WT + DSS, EpCAM^+/−^ and EpCAM^+/−^ + DSS groups.

To further evaluate the severity of the inflammation at molecular levels, the expression of the colonic abundant matrix metalloproteinases (MMPs) was subsequently checked. The transcriptional levels of Mmp2 (*p ≤ 0.001*), Mmp8 (*p < 0.05*) and Mmp12 (*p ≤ 0.001*) were all evidently higher in the EpCAM^+/−^ + DSS group than the WT, WT + DSS and EpCAM^+/−^ groups, and the mRNA levels of Mmp2 (*p < 0.05*) and Mmp8 (*p ≤ 0.01*) were also significantly increased in WT + DSS group compared to WT mice (Figure 2A). Moreover, the mRNA level of Mmp2 was noticeably higher in EpCAM^+/−^ group than the WT group (*p ≤ 0.01*) (Figure 2A). The protein levels of MMP7 (*p ≤ 0.001*) and MMP8 (*p < 0.05*) were all evidently higher in the EpCAM^+/−^ + DSS group than the WT, WT + DSS and EpCAM^+/−^ groups, and they are also significantly increased in the WT + DSS group compared to WT group (*p ≤ 0.01*) (Figures 2B,C). Furthermore, the protein level of MMP7 was significantly higher in EpCAM^+/−^ group than the WT group (*p < 0.05*), and MMP8 was also increased in EpCAM^+/−^ mice compared to the WT group (*p = 0.232*) although not significantly (Figures 2B,C). The increase of the expression of MMP2 and MMP7 in the colon indicated that the EpCAM^+/−^ mice are more sensitive to colitis than WT mice, and the elevation of the levels of MMP2, MMP7, MMP8 and MMP12 further confirmed that the DSS administration induced the severer inflammation in the colon of EpCAM^+/−^mice than the WT mice.

Furthermore, the activation of MAP kinases (MAPKs) was also tested through western blot. The levels of p-JNK, p-p38 and p-ERK1/2 were all significantly higher in the EpCAM^+/−^ + DSS group than the WT, WT + DSS and EpCAM^+/−^ groups (*p ≤ 0.001*), although the total proteins of JNK, p38 and ERK1/2 had no significant difference among 4 groups (*p = 0.192–0.909*) (Figures 2B,C). However, the phosphorylated proteins of JNK, ERK1/2 and p38 had no significant change in WT + DSS mice comparted to WT group (*p = 0.407–0.666*) (Figures 2B,C). The levels of p-JNK and p-ERK1/2 were also significantly upregulated in EpCAM^+/−^ mice compared to WT group (*p ≤ 0.01*) although the p-p38 showed no significant difference between EpCAM^+/−^ and WT groups (*p = 0.192*) (Figures 2B,C), indicating the increase of the sensitivity of EpCAM^+/−^ mice to colitis.

### 3.3 The heterozygous mutation of EpCAM aggravated the downregulation of tight junction components and pIgR in the colon of DSS-induced mice

**FIGURE 3:**
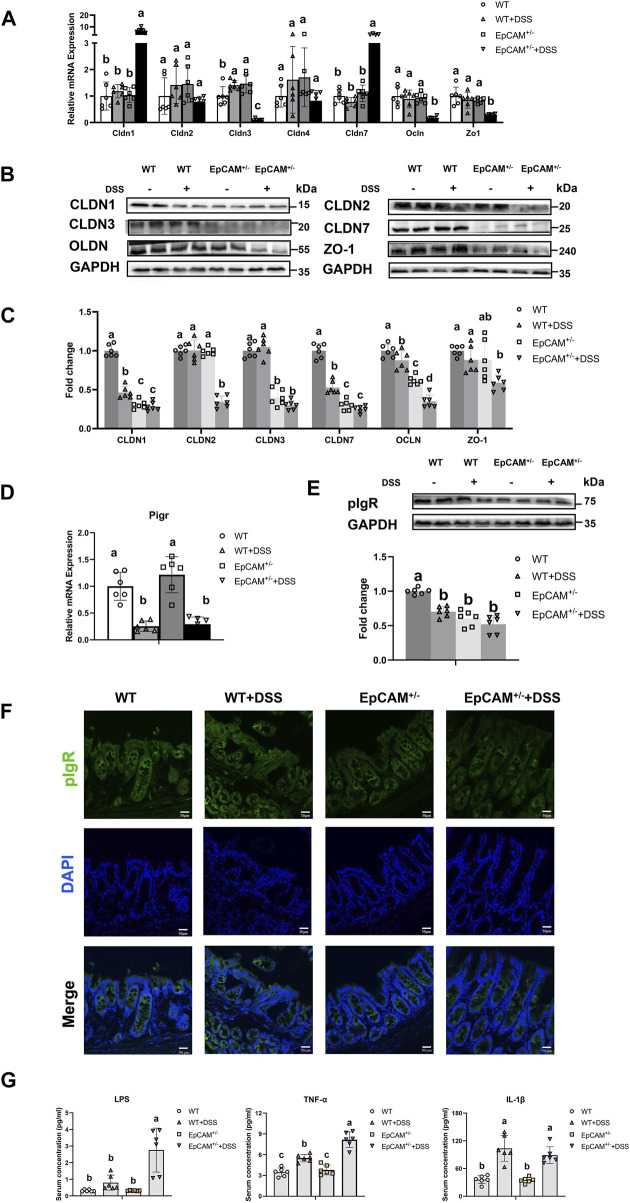
DSS stimulation downregulated the expression of genes for tight junction proteins and pIgR in the colon of EpCAM^+/−^ mice **(A)**. Graphs showed the qPCR results of Cldn1, Cldn2, Cldn3, Cldn4, Cldn7, Zo1 and Occludin in the colon of mice from WT, WT + DSS, EpCAM^+/−^ and EpCAM^+/−^ + DSS groups **(B)**. Representative images of the western blot results of CLDN1, CLDN2, CLDN3, CLDN7, OCLN and ZO-1 for the colon of mice from WT, WT + DSS, EpCAM^+/−^ and EpCAM^+/−^ + DSS groups **(C)**. Quantification data of the western blot results of CLDN1, CLDN2, CLDN3, CLDN7, OCLN and ZO-1 for the colon of mice from WT, WT + DSS, EpCAM^+/−^ and EpCAM^+/−^ + DSS groups **(D)**. The relative mRNA expression level of Pigr in the colon of mice from WT, WT + DSS, EpCAM^+/−^ and EpCAM^+/−^ + DSS groups **(E)**. The western blot results of pIgR for the colon of mice from WT, WT + DSS, EpCAM^+/−^ and EpCAM^+/−^ + DSS groups. Lower panel: Quantification data of the western blot results of pIgR for the colon of mice from WT, WT + DSS, EpCAM^+/−^ and EpCAM^+/−^ + DSS groups **(F)**. Representative images of immunofluorescence staining with antibodies to pIgR of frozen sections of colon from mice of WT, WT + DSS, EpCAM^+/−^ and EpCAM^+/−^ + DSS groups. DAPI was used for counter staining. Scale bar, 50 μm **(G)**. The concentrations of LPS, TNFα and IL-1β in the serum of mice from WT, WT + DSS, EpCAM^+/−^ and EpCAM^+/−^ + DSS groups.

To explore the related mechanism, the expression of the components of tight junctions including members of Claudin (Cldn) family, Zo-1 and Occludin (Ocln) was first tested. The transcriptional levels of Cldn1 and Cldn7 were evidently higher in the EpCAM^+/−^ + DSS group than the WT, WT + DSS, and EpCAM^+/−^ groups (*p ≤ 0.001*) (Figure 3A). However, DSS administration caused the significant decrease of CLDN1 and CLDN7 proteins in WT + DSS group compared to WT mice (*p ≤ 0.001*), and moreover, the protein levels of CLDN1 and CLDN7 were significantly lower in EpCAM^+/−^ and EpCAM^+/−^ + DSS groups than the WT and WT + DSS groups (*p < 0.05*) (Figures 3B,C). These results confirmed our previous report that EpCAM regulates the expression of Cldn1 and Cldn7 at the post-transcriptional level (Lei et al., 2012; Huang et al., 2018). There was no significant difference of the mRNA levels of Cldn2 and Cldn4 among the 4 groups (*p = 0.101–1.000*), (Figure 3A). However, the protein level of CLDN2 was noticeably decreased in the EpCAM^+/−^ + DSS group compared to the WT, WT + DSS and EpCAM^+/−^ groups (*p ≤ 0.001*) (Figures 3B,C). The DSS administration significantly upregulated the transcription of Cldn3 in WT mice (*p < 0.05*) but evidently downregulated it in EpCAM^+/−^ mice (*p ≤ 0.001*) (Figure 3A). At the protein level, the CLDN3 was significantly decreased in both EpCAM^+/−^ and EpCAM^+/−^ + DSS groups compared to mice from WT and WT + DSS groups (*p ≤ 0.001*) (Figures 3B,C). The mRNA levels of Zo1 and Ocln were noticeably lower in the EpCAM^+/−^ + DSS group than the WT, WT + DSS, and EpCAM^+/−^ groups (*p ≤ 0.001*) (Figure 3A). At the protein level, DSS administration significantly reduced the OCLN in both WT (*p* < *0.05*) and EpCAM^+/−^ mice (*p ≤ 0.001*), and the OCLN is significantly lower in EpCAM^+/−^ group than the WT group (*p ≤ 0.001*) (Figures 3B,C). Furthermore, the OCLN was significantly reduced in EpCAM^+/−^ + DSS group compared to the WT + DSS group (*p ≤ 0.001*) (Figures 3B,C). The ZO-1 protein was significantly lower in the EpCAM^+/−^ + DSS group than the WT (*p ≤ 0.001*) and WT + DSS groups (*p < 0.05*) (Figures 3B,C).

The expression of Pigr in the colon was also tested at both mRNA and protein levels. The administration of DSS significantly inhibited the transcription of Pigr in both WT and EpCAM^+/−^ mice (*p ≤ 0.01*) (Figure 3D). The protein level of pIgR was significantly lower in the WT + DSS, EpCAM^+/−^ and EpCAM^+/−^ + DSS groups than the WT mice (*p ≤ 0.001*), although the localization of pIgR had no evidently change in the IECs of them (Figures 3E,F).

Consistent with the decrease of tight junction proteins in the intestinal epithelium, the serum concentration of LPS was significantly increased in mice from EpCAM^+/−^ + DSS group compared to the WT (*p* < *0.05*), WT + DSS (*p ≤ 0.05*), EpCAM^+/−^ (*p* < *0.05*) groups (Figure 3G). The DSS administration caused the significant increase of the serum concentration of TNFα in both WT and EpCAM^+/−^ mice (*p ≤ 0.001*), and the serum concentration of TNFα was significantly higher in the EpCAM^+/−^ + DSS group than the WT + DSS group (*p ≤ 0.001*) (Figure 3G). The serum concentration of IL-1β was significantly increased in both WT and EpCAM^+/−^ mice after administration of DSS (*p ≤ 0.01*) (Figure 3G).

### 3.4 The heterozygous mutation of EpCAM caused the inflammation in the liver of DSS-induced mice

**FIGURE 4:**
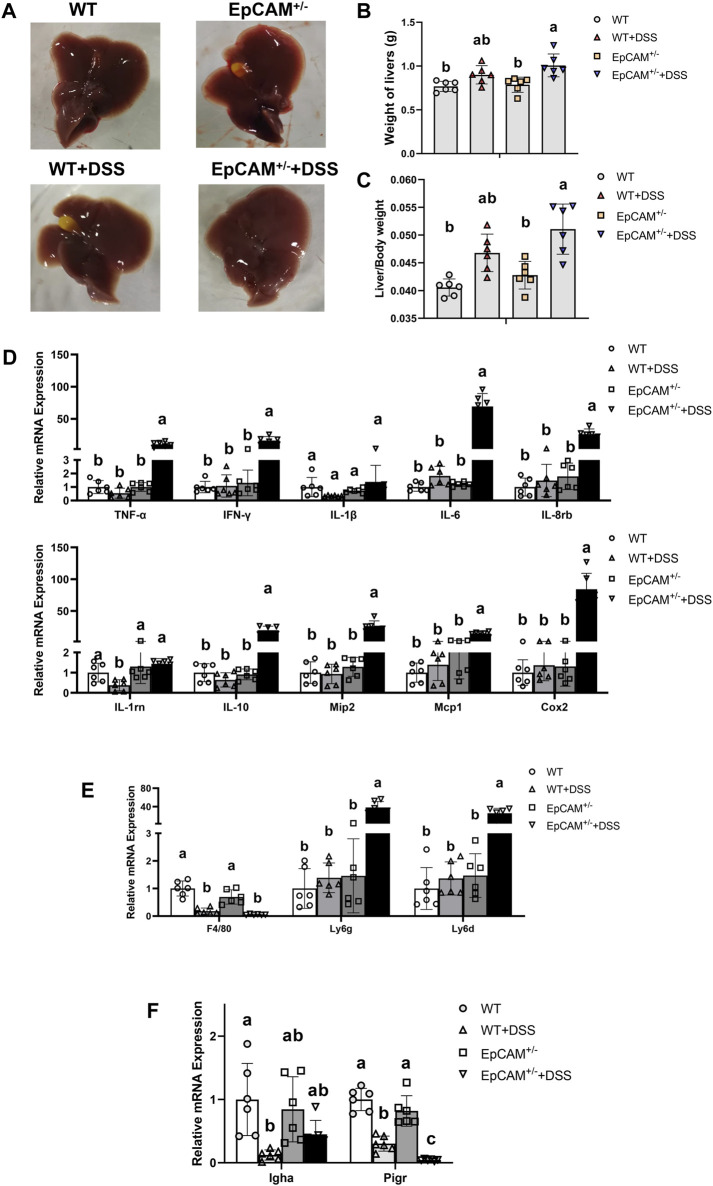
DSS stimulation caused serious inflammation in the liver of EpCAM^+/−^ mice **(A)**. Images of the liver of mice from WT, WT + DSS, EpCAM^+/−^ and EpCAM^+/−^ + DSS groups **(B)**. Graph showed the liver weight of mice from WT, WT + DSS, EpCAM^+/−^ and EpCAM^+/−^ + DSS groups **(C)**. Graph showed the liver index of mice rom WT, WT + DSS, EpCAM^+/−^ and EpCAM^+/−^ + DSS groups **(D)**. Graphs showed the qPCR results of TNFα, IFNγ, IL-1β, IL-6, IL-8rb, IL-1rn, IL-10, Mip2, Mcp1 and Cox2 in the liver of mice from WT, WT + DSS, EpCAM^+/−^ and EpCAM^+/−^ + DSS groups **(E)**. Relative mRNA levels of F4/80, Ly6g and Ly6d in the liver of mice from WT, WT + DSS, EpCAM^+/−^ and EpCAM^+/−^ + DSS groups **(F)**. Relative mRNA levels of Igha and Pigr in the liver of mice from WT, WT + DSS, EpCAM^+/−^ and EpCAM^+/−^ + DSS groups.

To confirm if the increased LPS would affected the hepatic tissues, the livers were next tested. The morphology of livers from the 4 groups showed no significant difference (Figure 4A). However, the DSS administration caused the significant increase of the liver weight (*p ≤ 0.001*) and liver index (*p < 0.05*) of EpCAM^+/−^ mice (Figures 4B,C). The mRNA levels of TNF-α, IFN-γ, IL-6, IL-8rb, IL-10, Mip2, Mcp1, and Cox2 were all significantly higher in the EpCAM^+/−^ + DSS group than the WT, WT + DSS, EpCAM^+/−^ groups (*p ≤ 0.01*) (Figure 4D).

The markers of inflammatory cells were subsequently checked via qPCR assay. The transcriptional level of F4/80 was significantly reduced in the DSS administrated both WT and EpCAM^+/−^ mice (*p ≤ 0.001*) (Figure 4E). The levels of Ly6g and Ly6d were all evidently higher in the EpCAM^+/−^ + DSS group than the WT, WT + DSS, EpCAM^+/−^ groups (*p ≤ 0.01*) (Figure 4E). The administration of DSS significantly decreased the transcription of Igha in WT mice (*p < 0.05*) (Figure 4F). Intriguingly, the transcriptional level of Pigr was significantly reduced in DSS-induced both WT and EpCAM^+/−^ mice (*p ≤ 0.001*), and it was also significantly lower in EpCAM^+/−^ + DSS group than the WT + DSS group (*p < 0.05*) (Figure 4F), indicating the translocation of IgA from blood to bile should be affected in the hepatic tissues of DSS administrated mice, especially in EpCAM^+/−^ + DSS group.

### 3.5 The DSS administration exacerbated the gut microbiota dysbiosis in mice with heterozygous mutation of EpCAM

**FIGURE 5:**
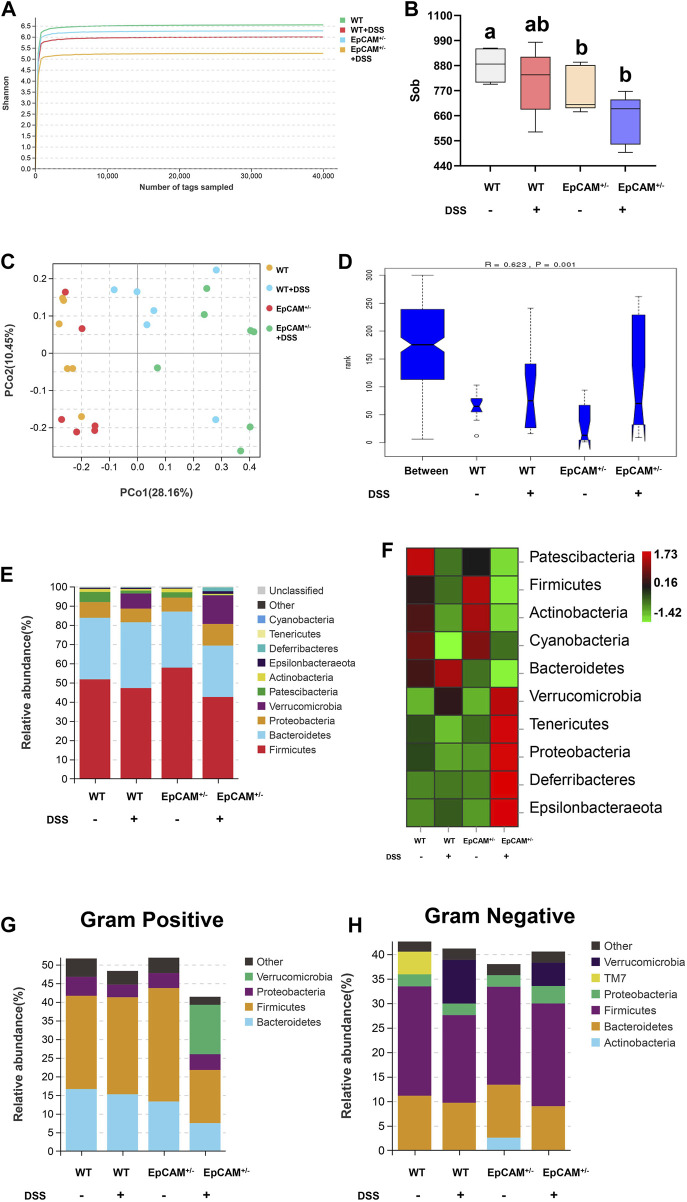
DSS stimulation altered the relative abundance of gut microbiota in both WT and EpCAM^+/−^ mice **(A)**. Shannon rarefaction curves for the WT, WT + DSS, EpCAM^+/−^ and EpCAM^+/−^ + DSS groups **(B)**. Graph showed the α diversity of the gut microbiota from the WT, WT + DSS, EpCAM^+/−^ and EpCAM^+/−^ + DSS groups **(C)**. The PCo analysis of the gut microbiota of mice from the WT, WT + DSS, EpCAM^+/−^ and EpCAM^+/−^ + DSS groups **(D)**. Analysis of similarity (ANOSIM) of the gut microbiota from the WT, WT + DSS, EpCAM^+/−^ and EpCAM^+/−^ + DSS groups. (**E, F)**. The relative abundance **(E)** and the heatmap **(F)** of the gut microbiota at phylum level in mice from the WT, WT + DSS, EpCAM^+/−^ and EpCAM^+/−^ + DSS groups. Different colors illustrated different flora. (**G, H)**. The relative abundance of gram negative **(G)** and gram positive **(H)** bacteria in the feces of mice from the WT, WT + DSS, EpCAM^+/−^ and EpCAM^+/−^ + DSS groups. Different colors illustrated different flora.

The 16S rDNA genes of the fecal microbiota from the 4 groups were sequenced. The Shannon curves of each group reached the saturation platforms (Figure 5A), demonstrating that the sequence coverage of samples from 4 groups was sufficient to represent the composition of the bacteria. The α diversity analysis results showed that the bacterial diversity of the EpCAM^+/−^ group was significantly reduced compared to WT group (*p < 0.05*), and the administration of DSS caused the reduction of the α diversity of both WT (*p = 0.219*) and EpCAM^+/−^ mice (*p = 0.089*) (Figure 5B). The PCo analysis (PCoA) indicated that samples from DSS treated and non-treated mice could be notably distinguished (Figure 5C). Analysis of similarity (ANOSIM) also confirmed the separation among the 4 groups (*R = 0.623, p = 0.01*) (Figure 5D).

The composition and changes of the gut microbiota at the phylum level were shown in Figures 5E,F. Compared with WT mice, the abundance of *Firmicutes* was increased but the abundance of *Bacteroidetes* was reduced in the EpCAM^+/−^ mice (Figures 5E,F). The DSS administration reduced the abundance of *Patescibacteria* in both WT and EpCAM^+/−^ mice, and the abundance of it was decreased in the EpCAM^+/−^ group compared to WT group (Figures 5E,F). The abundance of *Patescibacteria* in the EpCAM^+/−^ + DSS group was also reduced compared to the WT + DSS group (Figures 5E,F). The abundance of *Verrucomicrobia*, *Deferribacteres* and *Epsilonbacteraeota* was increased in both WT and EpCAM^+/−^ mice after administration of DSS, and the abundance of them was also increased in the EpCAM^+/−^ + DSS group compared to the WT + DSS group (Figures 5E,F). The abundance of *Tenericutes* and *Proteobacteria* was decreased in WT mice after administration of DSS, but the abundance of them was increased in EpCAM^+/−^ mice after administration of DSS (Figures 5E,F). The abundance of *Tenericutes* and *Proteobacteria* was also reduced in EpCAM^+/−^ group compared to the WT group, and the abundance of them was increased in the EpCAM^+/−^ + DSS group compared to the WT, WT + DSS and EpCAM^+/−^ groups (Figures 5E,F). Furthermore, the composition and changes of Gram-positive bacteria and Gram-negative bacteria from the feces was shown in Figures 5G,H.

**FIGURE 6:**
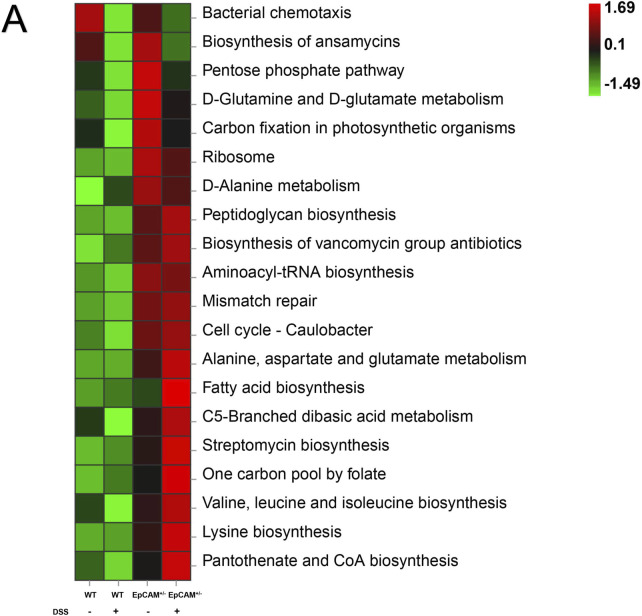
KEGG Assignments of the Gut Microbiota from the WT, WT + DSS, EpCAM^+/−^ and EpCAM^+/−^ + DSS groups **(A)**. KEGG analysis showed the top 20 altered pathways of the gut microbiota from the WT, WT + DSS, EpCAM^+/−^ and EpCAM^+/−^ + DSS groups.

The results of Kyoto Encyclopedia of Genes and Genomes (KEGG) pathway analysis showed the top 20 altered pathways (Figure 6A). The pathways of “Biosynthesis of ansamycins,” “Biosynthesis of vancomycin group antibiotics” and “Streptomycin biosynthesis” were all increased in EpCAM^+/−^mice compared to WT mice, and the increase of these pathways might be the important mechanism on the reduction of the bacterial diversity of the EpCAM^+/−^ group (Figure 6A). Moreover, the administration of DSS caused the further increase of “Biosynthesis of vancomycin group antibiotics” and “Streptomycin biosynthesis” pathways in the EpCAM^+/−^ mice, although the pathway of “Biosynthesis of ansamycins” was reduced in EpCAM^+/−^ mice with the treatment of DSS (Figure 6A).

The above results demonstrated the gut microbiota dysbiosis in the EpCAM^+/−^ mice and it was exacerbated by the DSS administration. Therefore, the dysbiosis of the gut microbiota might increase the sensitivity of EpCAM^+/−^ mice to DSS-induced IBD.

## 4 Discussion

Herein, the heterozygous mutation of EpCAM increased the sensitivity to colitis and exacerbated the dysbiosis of the gut microbiota in the DSS administrated mice because of the impaired tight junctions and the downregulated Pigr. The serious inflammation also occurred in the hepatic tissues from the DSS administrated heterozygotes of EpCAM mutant mice because of the increased serum LPS. Therefore, the normal level of EpCAM is essential to maintaining the immune balance of intestines and even liver, and the EpCAM mutation might be one of the potential risk factors for IBD.

The infiltration of immune cells, including macrophages, neutrophils and others, plays important roles in the occurrence of IBD (Chen et al., 2021; Lin et al., 2022; Yuan et al., 2022). F4/80 is the cell surface marker of macrophages (Meli et al., 2021; Niu et al., 2022), and the accumulation of F4/80^+^ macrophages has been detected in the ileum and colon of DSS-induced mice (Jiao et al., 2015). The Ly6G is the cell surface marker of neutrophils (Tanaka et al., 2015; Lei et al., 2022), and the frequency of the Ly6G^+^ neutrophils is higher in the severe inflammatory intestinal tissues of DSS-treated mice (Xie et al., 2018). In the present study, the levels of both F4/80 and Ly6g were all significantly higher in the colon of DSS-induced EpCAM^+/−^ mice than the WT + DSS group (Figure 1G). Hence, the infiltration of both macrophages and neutrophils should be increased in the DSS-induced EpCAM^+/−^ mice compared to the WT + DSS group. The corresponded results also could be observed in the colonic tissues from H&E staining (Figure 1D). Therefore, the more severe inflammation occurred in the colon of EpCAM^+/−^ mice than WT mice after the administration of DSS. The increase of both macrophages and neutrophils demonstrated that the colitis in the DSS-induced EpCAM^+/−^ mice showed the features of both chronic and acute inflammation. On the contrary, the increased Ly6g but reduced F4/80 in the colon from DSS-induced WT mice indicated that only the acute inflammation occurred (Figure 1G). IBD is characterized by the chronic inflammation in the gastrointestinal tract (Tanaka et al., 2015). Therefore, the EpCAM^+/−^ mice were more sensitive to the DSS-induced IBD than WT mice.

The MAPKs including JNK, ERK1/2 and p38 are associated with different stages of inflammatory process (Kaminska et al., 2010; Yeung et al., 2018; Tew et al., 2020; He et al., 2021). It was reported that DRAM1 aggravates the intestinal epithelium damage of IBD patients and mouse models through increasing the activation of JNK/c-Jun pathway, but the expression of DRAM1 shows no significant change in the intestinal tissues with acute inflammation (Zhang et al., 2021). The downregulation of EZH2 also causes IBD via activating JNK pathway both *in vivo* and *in vitro* (Lou et al., 2019). The increased p-ERK1/2 has been demonstrated to be the important mechanism of heat-stress induced IBD of pigs because it downregulates the expression of tight junction components in the intestinal epithelium (Yong et al., 2021). The increased MCT4 exacerbates the inflammation in IECs through enhancing the activation of ERK1/2-NFκB pathway (Wang et al., 2021). The endoplasmic reticulum (ER) stress in the IECs promotes IBD mediated by the activation of p38 signaling pathway, and the activated TLR4 promotes the inflammation of IBD through ER stress-p38 MAPK signal pathway (Hu et al., 2022; Long et al., 2022). IFN-γ has been detected to upregulate the expression of OTUD5 through p38-dependent mechanism to amplify the aberrant inflammatory cytokine response in IBD (Dinallo et al., 2022). In the present study, the activation of JNK, ERK1/2 and p38 signals was significantly increased in the EpCAM^+/−^ + DSS group but not in the WT + DSS group, and importantly the activation of JNK and ERK1/2 was also higher in EpCAM^+/−^ mice than WT mice (Figure 2C). Therefore, the susceptibility of EpCAM^+/−^ mice to IBD should be higher than the WT mice. At the same time, the higher concentration of LPS in the serum might promote the inflammation in the colon of mice from the EpCAM^+/−^ + DSS group through TLR4-ER stress-p38 pathway. The increased p-ERK1/2 might also exacerbates the impaired tight junctions in the colonic epithelium of the EpCAM^+/−^ + DSS group.

EpCAM plays important role on maintaining the tight junctions in the intestinal epithelium (Lei et al., 2012). In the present study, the protein levels of CLDN1, CLDN7 and OCLD were all significantly reduced in the EpCAM^+/−^ mice compared to WT mice (Figures 3B,C). The reduction of CLDN1 has also been detected in the colonic tissues of DSS-treated C57B/6 mice (Castro-Ochoa et al., 2019; Lee et al., 2021). The intestine-specific knockout of Cldn7 initiates the colonic inflammation because of the increase of the paracellular flux of the bacterial products from the intestinal lumen to lamina propria, such as LPS, peptidoglycan and N-formyl-L-methionyl-L-leucyl-L-phenylalanine (Tanaka et al., 2015). Therefore, the reductions of CLDN1 and CLDN7 in the colonic tissues might be one of the mechanisms of the increase of the susceptibility to IBD of the EpCAM^+/−^ mice. The reductions of CLDN1 and CLDN7 might causes the increase of the paracellular flux of the bacterial products from the intestinal lumen into blood of EpCAM^+/−^ mice.

EpCAM has important function on keeping the normal level of pIgR in the intestinal epithelium (Lei et al., 2022). Here, there was only around 50% of pIgR proteins in the colon of EpCAM^+/−^ mice compared to the WT mice (Figures 3E,F). The decrease of pIgR has been confirmed in the colonic mucosa of both Crohn’s disease (CD) and UC patients (Bruno et al., 2015). Furthermore, the somatic mutation of PIGR in the colon has been considered as one of the causal roles in the pathogenesis of IBD in patients (Olafsson et al., 2020). Therefore, the reduction of pIgR in the colonic tissues might be another mechanism on increasing the susceptibility to IBD for the EpCAM^+/−^ mice. Since IgA and IgM are transported into the intestinal lumen by pIgR to impair the pathogen colonization (Lokken-Toyli et al., 2021), the reduction of pIgR in the colonic tissues might cause the dysbiosis of the gut microbiota in the EpCAM^+/−^ mice.

For the composition of the gut microbiota, the increase of the abundance of *Firmicutes* and the decrease of the abundance of *Bacteroidetes* cause the higher ratio of *Firmicutes*/*Bacteroidetes* at the phylum level in the EpCAM^+/−^ mice than WT mice (Figures 5E,F). The increase of the ratio of *Firmicutes*/*Bacteroidetes* has been considered a dysbiotic pattern contributed to various diseases and it is correlated with the increase of the intestinal paracellular permeability (Muntjewerff et al., 2021; Wang S. et al., 2022). The abundances of *Patescibacteria* and *Proteobacteria* are significantly reduced in the gut microbiota of diarrheic horses compared with the healthy horses (Li Y. et al., 2022), and both of these two phyla were also reduced in the EpCAM^+/−^ mice compared to WT mice (Figures 5E,F). On the contrary, the relative abundance of *Verrucomicrobia* is dramatically increased in the gut microbiota of diarrheic horses (Li Y. et al., 2022). It was also reported that one of the major risk factors of IBD, sphingosine kinases (SphKs), can increase the *Verrucomicrobia* in the gut microbiota (Miao et al., 2022). In the present study, the DSS treatment caused the significant increase of the abundance of *Verrucomicrobia* in both WT and EpCAM^+/−^ mice, and it was higher in the EpCAM^+/−^ + DSS group than the WT + DSS group (Figures 5E,F). Hence, the genetic background of EpCAM^+/−^ mice caused the alteration of the gut microbiota, which increased the susceptibility to IBD.

The serum concentration of LPS was significantly increased in the EpCAM^+/−^ + DSS group because of the impaired tight junctions in the colonic epithelium (Figure 3G). The increased LPS in the blood has been demonstrated to cause the liver injury in DSS-induced mice (Guan et al., 2022). The high level of LPS in the serum also causes the hepatocyte necrosis and even cholestasis (Li T. et al., 2022). In the present study, the acute inflammation was occurred in the liver of DSS-induced EpCAM^+/−^ mice marked by the significant increase of Ly6g but the significant decrease of F4/80 in the hepatic tissues, but the liver of DSS-induced WT mice was still not significantly affected (Figure 4E). Hence, the liver of EpCAM^+/−^ mice might be also more sensitive to the DSS stimulation than WT mice. Importantly, the administration of DSS significantly downregulated the expression of Pigr at transcriptional level in the liver, especially in EpCAM^+/−^ mice (Figure 4F). The concentration of IgA should be reduced in the bile, and the transport of IgA from liver to the intestinal lumen might also be decreased. The reduction of the content of IgA in bile might aggravate the dysbiosis of gut microbiota in EpCAM^+/−^ mice.

**FIGURE 7:**
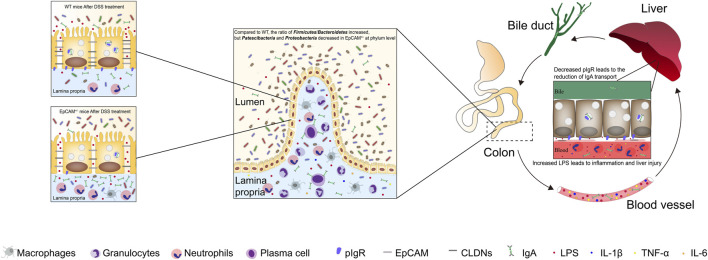
The heterozygous mutation of EpCAM increased the susceptibility to colitis, gut microbiota dysbiosis and liver injury of mice. The decreased EpCAM in the colonic tissues of the heterozygotes of EpCAM mutant mice caused the downregulation of the proteins of CLDN1, CLDN7, OCLD and pIgR, and then the intestinal paracellular permeability increased and the dysbiosis of gut microbiota occurred in the EpCAM^+/−^ mice. Therefore, the paracellular flux of LPS from the intestinal lumen to blood also increased in EpCAM^+/−^ mice. The higher concentration of LPS in the blood induced the occurrence of inflammation and the downregulation of Pigr in the hepatic tissues, and then the transport of IgA from liver to the intestines was also reduced to exacerbate the gut microbiota dysbiosis of EpCAM^+/−^ mice with the stimulation of DSS. Finally, it led to the vicious circle between the colitis, gut microbiota dysbiosis and liver injury of EpCAM^+/−^ mice if they are stimulated with environmental factors to occurrence of the colonic inflammation.

In conclusion, the heterozygous mutation of EpCAM increased the susceptibility to colitis, gut microbiota dysbiosis and liver injury of mice (Figure 7). The decreased EpCAM caused the downregulation of CLDN1, CLDN7, OCLD and pIgR at the post-transcriptional level, and then the intestinal paracellular permeability increased and the dysbiosis of gut microbiota occurred, the paracellular flux of LPS from the intestinal lumen to blood also increased. The higher concentration of LPS in the blood induced the occurrence of inflammation and the downregulation of Pigr in the liver, and then reduced the transport of IgA from liver to the intestines to exacerbate the gut microbiota dysbiosis. Finally, it led to the vicious circle between the colitis, gut microbiota dysbiosis and liver injury of EpCAM^+/−^ mice.

## Data Availability

The data presented in this study are deposited in the SRA repository, accession number PRJNA1144604.
