# Supplementary material for: The downregulation of tight junction proteins and pIgR in the colonic epithelium causes the susceptibility of EpCAM+/− mice to colitis and gut microbiota dysbiosis
Source: Front Mol Biosci. 2024 Aug 12;11:1442611. doi: 10.3389/fmolb.2024.1442611 (PMC11345229; doi:10.3389/fmolb.2024.1442611)
Supplement: Supplementary file 1 [file Table1.docx]

Table S1. Primer sequences of genes for qPCR

| Genes | Forward (from 5’to 3’) | Reverse (from 5’to 3’) |
| --- | --- | --- |
| Cldn1 | ACTGTGGATGTCCTGCGTTT | TCATGCCAATGGTGGACACA |
| Cldn2 | CAGAGTGGCTGTAGTGGGTG | AGTTGGTACGATTGCCCTGG |
| Cldn3 | CCCTCATCGTGGTGTCCATC | CGTCTCGTCTTGTACGCAGT |
| Cldn4 | AGCAACGACAAGCCCTACTC | CAGAGTGGCCCACCTTACAC |
| Cldn7 | GGCTTTTCAATGGCCATGCT | GCTGTGATGATGTTGTCGCC |
| Cox2 | TGAGTGTGGGATTTGACCAG | TGTGTTTGGAGTGGGTTTCA |
| F4/80 | GTGACTCACCTTGTGGTCCT | TTGCATGTTCAGGGCAAACG |
| GAPDH | TCATCATACTTGGCAGGTTTC | GCATCCACTGGTGCTGCC |
| IFN-γ | GGTCAACAACCCACAGGTCC | CAGCGACTCCTTTTCCGCTT |
| Igha | ACCGTAAACTTCCCACCTGC | GGACGGCGTTAGAGTCATGT |
| IL-10 | GGTTGCCAAGCCTTATCGGA | GCCTTGTAGACACCTTGGTCTT |
| IL-1rn | CTCGGGATGGAAATCTGCTGG | GACTTGGCACAAGACAGGCA |
| IL-1β | GCCACCTTTTGACAGTGATGAG | AAGGTCCACGGGAAAGACAC |
| IL-6 | CTCCCAACAGACCTGTCTATAC | CCATTGCACAACTCTTTTCTCA |
| IL-8rb | TCTTCCAGTTCAACCAGCC | ATCCACCTTGAATTCTCCCATC |
| Ly6d | CACTTCGATGTCACGTGTGC | GACCAGCCTCTCGTTGCATA |
| Ly6g | CAACACAACTACCTGCCCCT | AACCAGGCTGAACAGAAGCA |
| Mcp-1 | TTTTTGTCACCAAGCTCAAGAG | TTCTGATCTCATTTGGTTCCGA |
| Mip-2 | GGTTGACTTCAAGAACATCCAG | TTGAGAGTGGCTATGACTTCTG |
| Mmp2 | ACTTTGAGAAGGATGGCAAGTA | CTTCTTATCCCGGTCATAGTCC |
| Mmp3 | TGTGGTTGTGTGCTCATCCT | CCTGTCATCTCCAACCCGAG |
| Mmp8 | CCAAGCATGTTCCCAGGAGT | TTGTCCTCATAGGGTGCGTG |
| Mmp12 | TGTACAGCATCTTAGAGCAGTG | TATGTAGTCTACATCCTCACGC |
| Mmp13 | CCCCTTCCCTATGGTGAT | TCAACTGTGGAGGTCACT |
| Mmp15 | AAGCTGGGCTGGTACAACTC | ATCTGCGTCGAAATGGGTGT |
| Occludin | AAAGTCCACCTCCTTACAGACC | ACAGGCAAATATGGCGATGC |
| Pigr | CAGATACAAGAGATCAAGCCGA | TAGCTTCTGATTGAAACTCGGT |
| TNF-α | CTCCTCACCCACACCATCA | GGAAGACCCCTCCCAGATAG |
| Zo1 | GTCCCTGTGAGTCCTTCAGC | GACCAACCGTCAGGAGTCAT |

Table S2. Information of antibodies used for western blot

| Antibodies | Species | Cat. No. | Company | Dilutions |
| --- | --- | --- | --- | --- |
| Anti-Mouse | donkey | ab205724 | Abcam | 1/2000 |
| Anti-Rabbit | goat | SA00001-2 | Proteintech | 1/2000 |
| Anti-Rat | donkey | ab102182 | Abcam | 1/2000 |
| CLDN1 | rabbit | ab180158 | Abcam | 1/1000 |
| CLDN2 | rabbit | ab53032 | Abcam | 1/1000 |
| CLDN3 | rabbit | ab214487 | Abcam | 1/1000 |
| CLDN7 | rabbit | ab27487 | Abcam | 1/1000 |
| ERK1/2 | rabbit | ab184699 | Abcam | 1/1000 |
| GAPDH | rabbit | 14C10 | Cell Signaling Technology | 1/1000 |
| JNK | rabbit | 9252 | Cell Signaling Technology | 1/1000 |
| MMP7 | rabbit | ab38996 | Abcam | 1/1000 |
| MMP8 | rabbit | ab81286 | Abcam | 1/1000 |
| OCLN | rabbit | 81829 | Proteintech | 1/1000 |
| p38 | rabbit | ab38238 | Abcam | 1/1000 |
| p-ERK1/2 | rabbit | 9101 | Cell Signaling Technology | 1/1000 |
| pIgR | rat | ab170321 | Abcam | 1/1000 |
| p-JNK | mice | 9255 | Cell Signaling Technology | 1/1000 |
| p-p38 | rabbit | ab38238 | Abcam | 1/1000 |
| ZO-1 | rabbit | 104354 | Proteintech | 1/1000 |
